# Accurate Structures and Spectroscopic Parameters of Phenylalanine and Tyrosine in the Gas Phase: A Joint Venture of DFT and Composite Wave-Function Methods

**DOI:** 10.1021/acs.jpca.3c01174

**Published:** 2023-04-13

**Authors:** Vincenzo Barone, Marco Fusè

**Affiliations:** † Scuola Normale Superiore di Pisa, Piazza dei Cavalieri 7, 56126 Pisa, Italy; ‡ DMMT-sede Europa, Università di Brescia, Viale Europa 11, 25121 Brescia, Italy

## Abstract

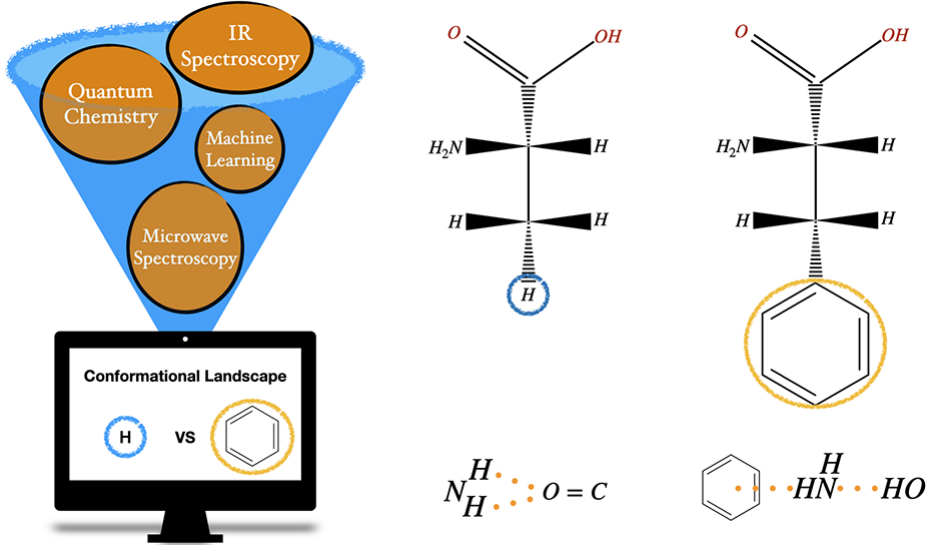

A general strategy for the accurate computation of conformational and spectroscopic properties of flexible molecules in the gas phase is applied to two representative proteinogenic amino acids with aromatic side chains, namely, phenylalanine and tyrosine. The main features of all the most stable conformers predicted by this computational strategy closely match those of the species detected in microwave and infrared experiments. Together with their intrinsic interest, the accuracy of the results obtained with reasonable computer times paves the route for accurate investigations of other flexible bricks of life.

## Introduction

Amino acids represent central targets for accurate experimental and theoretical studies because their rich conformational landscape is tuned by the competition among different kinds of noncovalent interactions.^1,2^ While zwitterionic forms of amino acids are found in crystals^3^ and aqueous solutions,^4^ neutral forms are observed in the gas phase^5−7^ and in low-temperature inert matrices.^8,9^ In the latter case, van der Waals forces between the sample and inner gas molecules have usually a negligible effect on spectroscopic parameters,^10^ so high-resolution spectroscopic studies both in the gas phase (microwave, MW) and in inert matrices (infrared, IR) allow an unbiased disentanglement of intrinsic stereoelectronic effects without any strong perturbation from the environment.

Systematic investigations have revealed that the natural amino acids containing simple nonpolar side chains present two dominant conformers (usually referred to as type I and type II), stabilized by intrabackbone hydrogen bonds.^7,11,12^ On the other hand, the conformational landscape of natural amino acids with polar side chains is much richer due to the synergy or competition between intrabackbone and backbone–(side chain) hydrogen bonds.^13−16^ Here we will analyze in detail the situation for prototypical amino acids containing aromatic side chains (phenylalanine (Phe) and tyrosine (Tyr)), which can show an additional kind of noncovalent interaction between polar hydrogen atoms of the backbone and the π-system of the side chain.

Phenylalanine and tyrosine have been studied in the gas phase by high-resolution laser-induced fluorescence (LIF), hole-burning UV–UV, ion dip IR–UV, resonance-enhanced multiphoton ionization (REMPI) spectroscopy,^6,17−22^ and, more recently, by microwave (MW) spectroscopy in combination with supersonic jets.^23,24^ Phenylalanine has been studied also by matrix-isolation infrared spectroscopy.^10^ These experiments have been interpreted in terms of a variable number of low-energy conformers due to the presence of effects (e.g., fast relaxation of some structures to more stable counterparts in the presence of low energy barriers and photodissociation of some products) playing a different role under different experimental conditions.^20,25,26^

Accurate quantum chemical (QC) computations can help to solve this kind of problems,^27−29^ but the size of the systems and the number of different structures to be analyzed prevent a systematic use of very accurate but very expensive state-of-the-art QC methodologies.^30−32^ Furthermore, an effective exploration of flat potential energy surfaces (PES) cannot be performed by straightforward systematic searches and/or local optimizations.^33,34^ We have recently developed an integrated computational approach combining different QC methods driven by machine learning (ML) tools for the effective exploration of conformational PES and the successive refinement of the most significant stationary points.^35−37^ In this framework, once a suitable panel of low-energy minima has been defined, accurate structures are obtained by refining the optimized geometries by a linear regression approach^38,39^ and accurate relative energies are computed by reduced-scaling composite methods.^40−45^ The zero point energies (ZPEs) and thermal contributions leading from electronic to free energies are evaluated by parameter-free anharmonic approaches rooted in the second order vibrational perturbation theory (VPT2)^46−53^ for sufficiently stiff degrees of freedom and proper treatment of hindered rotations.^54,55^ Finally, accurate spectroscopic parameters of the energy minima with non negligible populations under the experimental conditions of interest are computed.^56^

This procedure has been recently validated for several amino acids containing polar side chains.^57−59^ Here we tackle the problem of aromatic side chains analyzing both the rotational and vibrational spectra of the prototypical phenylalanine and tyrosine amino acids. In fact, previous studies of these amino acids employed QC methods of limited accuracy, payed marginal attention to the geometrical parameters and neglected vibrational corrections beyond the standard rigid rotor harmonic oscillator (RRHO) model. However, these limitations hamper any *a priori* prediction of the spectroscopic outcome, allowing at most its *a posteriori* interpretation in terms of the agreement between experimental and computed spectroscopic parameters for a predefined number of conformers. Our approach allows, instead, an unbiased comparison with spectroscopic results thanks to the accuracy of the computational results, which provide mean unsigned errors (MUEs) within 10 MHz for rotational constants and 10 cm^–1^ for both relative energies and vibrational frequencies.^41,60^

## Methods

A first characterization of low-energy conformers is performed at the B3LYP/jun-cc-pVDZ level,^61,62^ also including Grimme’s D3BJ dispersion corrections.^63^ In the following this computational model is referred to simply as B3 and is used also for the computation of anharmonic contributions. The geometries and harmonic force fields of conformers lying within a predefined energy range are then refined by the revDSD-PBEP86-D3BJ/jun-cc-pv(T+d)Z model^64−67^ (hereafter rDSD), which provides excellent conformational landscapes,^30,68,69^ geometries,^39^ dipole moments,^70^ and spectroscopic parameters.^56,71^ In the present context, we will analyze in detail only conformers not involved in fast relaxation processes and with a relative free energy below 620 cm^–1^ at room temperature (i.e., a relative population of about 5% when only the considered conformer and the absolute free energy minimum are taken into account, since *kT*/*hc* = 207 cm^–1^).

The typical MUEs of rDSD bond lengths and valence angles (0.3%)^39^ are sufficient to obtain accurate relative electronic energies and vibrational spectra of different conformers, but the situation is different for the leading terms of MW spectra, namely, rotational constants of the vibrational ground state (*B*_0_^*i*^, where *i* refers to the inertial axes *a*, *b*, *c*). These parameters include vibrational corrections (Δ*B*_vib_^*i*^) in addition to equilibrium rotational constants (*B*_*e*_^*i*^).^72^ In the framework of the VPT2 approximation,^73^ the ground-state rotational constants can be expressed as
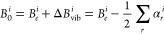
1where the α_*r*_’s are the vibration–rotation interaction constants and the sum runs over all *r* vibrational modes (for details, see refs (31), (74), and (75)). With Δ*B*_vib_^*i*^ contributions typically well below 1% of the corresponding *B*_*e*_^*i*^ rotational constants,^76^ they can be safely determined at affordable levels of theory (B3 in the present context), which deliver typical errors within 10% (i.e., less than 0.1% of typical rotational constants).^31,77^ On the other hand, inclusion of vibrational corrections is not warranted if the errors on the computed rotational constants are not much lower than 1% (10 MHz for a constant of 1000 MHz). Therefore, equilibrium rotational constants require very accurate geometrical parameters, which can be obtained only with state-of-the-art QC methods,^78−81^ although reasonable relative errors (typically 0.4–0.5%) are obtained at the rDSD level.^39^ We have recently shown that the systematic nature of the errors permits to improve significantly the rDSD geometrical parameters, and thus equilibrium rotational constants, by a linear regression approach (hereafter LRA).^39,82,83^ In this model, the computed geometrical parameters (*r*_*comp*_) are corrected for systematic errors by means of scaling factors (*a*) and offset values (*b*) depending on the nature of the involved atoms and determined once for ever from a large database of accurate semiexperimental (SE) equilibrium geometries:^39,59,83^
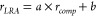
2

Since at least one ^14^N quadrupolar nucleus is present in all amino acids, nuclear quadrupole coupling constants (χ_*ii*_, with *i* referring to the inertia axis *a*, *b*, or *c*) are important for accurate predictions of rotational spectra because they determine a splitting of the rotational transitions, which generates the so-called hyperfine structure.^78,84^ Furthermore, the components of dipole moments (μ_*i*_) determine the intensities of rotational transitions.^74,84^ Several studies have shown that dipole moments and quadrupole coupling constants can be computed with sufficient accuracy at the rDSD level.^59,70^ On the other hand, accurate electronic energies can be obtained by single-point energy evaluations at rDSD geometries using composite wave function methods rooted in the coupled cluster (CC) ansatz.^85^ In particular, the CC model including single, double, and perturbative estimate of triple excitations (CCSD(T))^86^ is employed here taking also into account complete basis set (CBS) extrapolation and core valence (CV) correlation. The starting point of the family of “cheap” schemes (ChS) developed in the last years^28,36,40,41^ is a frozen core (fc) CCSD(T) computation in conjunction with a (partially augmented) triple-ζ basis set.^62,66,87^ Then, in analogy with the correlation consistent composite approach (ccCA),^88,89^ CBS and CV terms are computed with good accuracy and negligible additional cost by means of second order Møller–Plesset perturbation theory (MP2).^90^ In particular, the CBS extrapolation by the standard *n*^–3^ two-point formula^91^ employs MP2/jun-cc-pV(X+d)Z energies with X = T and Q, whereas the CV contribution is incorporated as the difference between all-electron (ae) and fc MP2 calculations, both with the cc-pCVW(T+d)Z basis set.^92^ Replacement of conventional methods by the explicitly correlated (F12) variants^93,94^ leads to our current standard version of the approach, which is referred to as junChS-F12.^42,58,60^ Comparison with the most accurate results available for a panel of representative noncovalent complexes provided an average absolute error smaller than 10 cm^–1^.^41,42,60^

The relative stability of different conformers is determined by the corresponding relative enthalpy at 0 K (Δ*H*_0_^°^) or free energy (Δ*G*°) at a temperature depending on the experimental conditions. The vibrational contributions to thermodynamic functions of stiff degrees of freedom are computed in the framework of second-order vibrational perturbation theory (VPT2),^46,48,50,73^ employing rDSD harmonic and B3 anharmonic contributions. The same computations provide also anharmonic IR spectra.^53,95,96^ Low-frequency contributions are, instead, taken into account by means of the quasi-harmonic (QH) approximation.^54,97^

All the computations have been performed with the Gaussian package^98^ except the junChS-F12 and QH ones, which have been performed with the help of the Molpro^99^ and GoodVibes^97^ software, respectively.

## Results and Discussion

**Figure 1:**
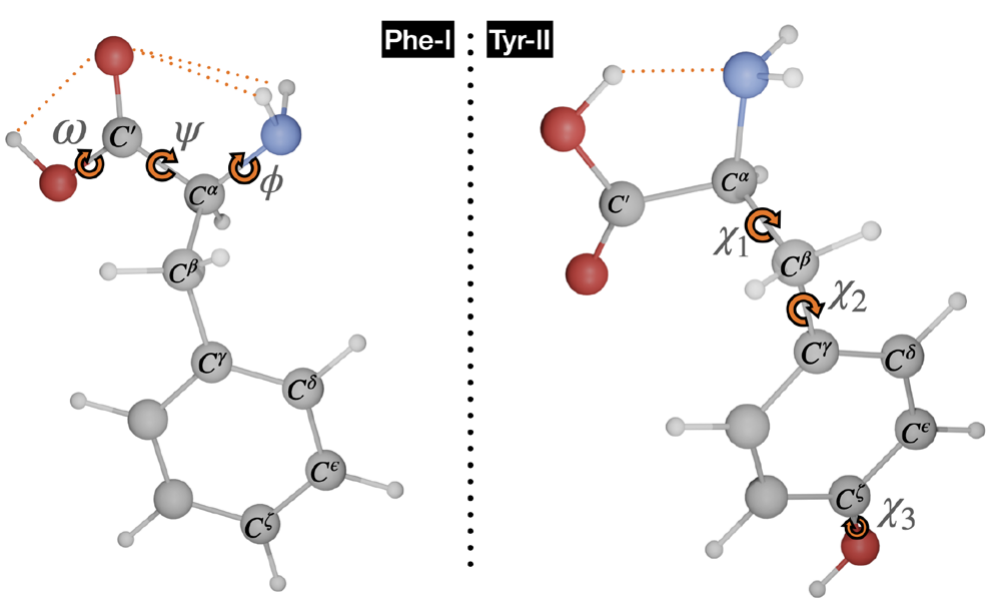
Structure and dihedral angles of phenylalanine (conformer of type I) and tyrosine (conformer of type II).

The conformational landscape of Phe and Tyr is ruled by two soft degrees of freedom in the backbone (ϕ = H–N–C^α^–C′ and ψ = N–C^α^–C′–O(H) dihedral angles) and two others in the side chain (χ_1_ = NC^α^–C^β^–C^γ^ and χ_2_ = C^α^–C^β^–C^γ^–C^δ^ dihedral angles) (see Figure 1). However, the nonplanarity of the NH_2_ moiety suggests to replace the ϕ dihedral angle with ϕ′ = LP–N–C^α^–C′ = ϕ + 120°, where LP is the nitrogen lone-pair perpendicular to the plane defined by the two amine hydrogens and the C^α^ atom. The customary *c*, *g*, *s*, *t* labels are then used to indicate the cis, gauche, skew, and trans conformations determined by ϕ′, ψ, χ_1_, and χ_2_ dihedral angles.

Only planar conformations are allowed for the carboxylic moiety of both amino acids (ω = C^α^–C′–O–H = 0° or 180°) and the phenol group of tyrosine (χ_3_ = C^ϵ^–C^ζ^–O–H = 0° or 180°). In the former case ω = 0° is strongly preferred (due the formation of a weak hydrogen bridge with the carbonyl oxygen) except when the oxidryl hydrogen is involved in stronger hydrogen bonds with other acceptor groups. In the case of χ_3_ the two nonequivalent arrangements of the phenol OH moiety are labeled in the following as N and C with reference to the H(O) placement on the same side of the backbone N or C′ atom, respectively.

**Figure 2:**
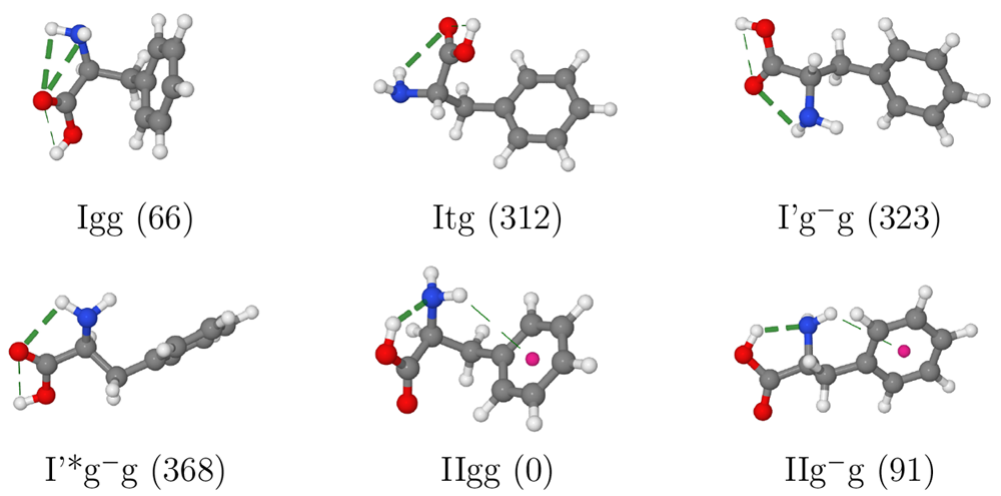
Conformers of phenylalanine with the computed relative free energies at room temperature (in cm^–1^) given in parentheses. H-bonds are highlighted by dashed lines.

The exploration of the conformational landscape of phenylalanine provided several low-energy conformers stabilized by the formation of hydrogen bonds, classified as type I (bifurcated NH_2_···O=C, ϕ′ ≈ 180°, ψ ≈ 180°, ω ≈ 180°), I′ (HNH···O=C, ϕ′ ≈ 90°, ψ ≈ 180°, ω ≈ 180°), and II (N···HO, ϕ′ ≈ 0°, ψ ≈ 0°, ω ≈ 0°).^100^ Some conformers in which the O(H) oxygen of the carboxyl group forms a bifurcated hydrogen bond with the NH_2_ moiety (type III, ϕ′ ≈ 180°, ψ ≈ 0°, ω ≈ 180°) have been also found, but they can easily relax to more stable I conformers by rotation of 180° around ψ. In fact, III conformers have been observed in MW spectra only in very special situations.^101^ Also some conformers of type I relax to more stable structures (still of type I) through rotation around the χ_1_ dihedral angle. As a consequence, we are left with the six conformers shown in Figure 2, with the two I′ conformers corresponding to the nonequivalent g and g^–^ orientations of the ϕ′ dihedral angle (see Table 1).

**Table 1:** rDSD Relative Electronic Energies (Δ*E*_*rDSD*_), Harmonic Zero Point Energies (ΔZPE_*H*_), Thermal Contributions (ΔTh_*H*_), and Quasi-Harmonic Corrections to Free Energies (*T*Δ*S*_*QH*–*H*_), Together with Differences between junChS-F12 and rDSD Electronic Energies (ΔChS) and B3 Anharmonic Corrections to ZPEs (ΔZPE_*anh*–*H*_) for the Low-Lying Conformers of Phenylalanine^a^

label	Δ*E*_*rDSD*_	ΔChS	ΔZPE_*H*_	ΔTh_*H*_	ΔZPE_*anh*–*H*_	TΔ*S*_*QH*–*H*_	Δ*G*°^b^	ϕ′	ψ	ω	χ_1_	χ_2_
Igg	317.3	–4.6	–152.1	–142.2	49.4	–2.2	65.6	–178.4	174.3	–178.9	62.6	89.6
Itg	596.9	36.6	–180.8	–236.2	41.3	54.2	312.0	173.5	129.7	177.4	179.9	81.4
I′g^–^g	660.4	–11.0	–190.7	–206.1	43.1	26.9	322.6	–74.4	151.9	176.2	–60.4	99.1
I′*g^–^g	762.1	–47.7	–212.8	–210.3	73.0	3.5	367.8	80.2	–169.2	–177.2	–61.9	93.9
IIgg	0.0	0.0	0.0	0.0	0.0	0.0	0.0	–26.1	12.2	–2.6	52.7	81.0
IIg^–^g	176.9	7.5	–31.6	–92.8	31.6	–0.9	90.7	33.8	–18.0	4.0	–63.2	103.3

a Best estimates of relative free energies at room temperature (Δ*G*°) and dihedral angles optimized at the rDSD level (ϕ′, ψ, ω, χ_1_, and χ_2_) are also given. All the energetic quantities are in cm^–1^ and the angles in degrees.

b Sum of columns 2–7.

The structural and energetic characteristics of those six low-energy conformers are reported in Table 1, whereas the different contributions to the final electronic energies are given in Table S1 and the main spectroscopic parameters are given in Tables S2 and S3. The first noteworthy feature is the remarkable accuracy of rDSD conformational energies (MUE and maximum unsigned error (MAX) of 21.5 and 47.7 cm^–1^ with respect to the junChS-F12 reference). Although rDSD electronic energies are not directly used to estimate relative stabilities, this finding gives further support to the use of rDSD geometries and harmonic force fields. Different staggered conformations are populated for χ_1_, which determines the relative position of the phenyl ring with respect to the amino acid backbone (see Figure 1). On the other hand, all low-energy conformers show a C^α^–C^β^ bond nearly perpendicular to the plane of the phenyl ring (χ_2_ ≈ 90°) and this feature has been confirmed experimentally by ENDOR spectroscopy for spin-labeled l-phenylalanine.^102^ Interestingly, the same region of χ_2_ is dominantly populated and characteristic of Phe residues in proteins.^103^

Contrary to the usual situation for aliphatic amino acids,^59^ conformers of type II are more stable than their types I and I′ counterparts in spite of a less favorable orientation of the OH group in the carboxyl moiety. The increased stability of these conformers is related to the existence of a “daisy chain” sequence of interactions involving aromatic π electrons, together with the NH_2_ and OH groups of the backbone.^19^ Zero-point and thermal effects stabilize all conformers with respect to the IIgg absolute energy minimum, with this effect being particularly significant for conformers of types I and I′. As a consequence, conformer Igg becomes the second most stable form, with a relative free energy (with respect to IIgg) marginally lower than that of the IIg^–^g conformer. Inclusion of anharmonic contributions in ZPEs is needed for obtaining quantitative results, but it does not alter the stability order of the different conformers. Finally, the main effect of the QH corrections is to reduce the overestimation of entropic contributions produced by the harmonic oscillator approximation, with the consequent excessive stabilization of all conformers with respect to the IIgg species. The final results suggest that Igg and IIg^–^g conformers have comparable populations (about 23% each at room temperature) only slightly lower than that of the IIgg conformer (about 33% at room temperature), whereas the other three conformers have equilibrium populations around 7% each at room temperature and their unequivocal characterization could be, therefore, quite difficult.

The rotational constants are very sensitive to the value of the χ_1_ dihedral angle, with g conformers showing values around 1650, 650, and 450 MHz, whereas g^–^ conformers show values around 2450, 450, and 400 MHz (see Tables 2 and S2). On the other hand, much larger dipole moments are computed for conformers of type II with respect to those of type I, irrespective of the orientation of the side chain (see Table S3). These general trends were used to support the assignment of experimental MW spectra^23^ and are not very sensitive to the level of the QC computations. However, comparison of Table 2 (and Table S2) with the results of previous investigations^23^ shows that our computational approach strongly improves the quantitative agreement between the computed and experimental rotational constants of the detected conformers concerning both the parent species and the ^15^N isotopomers (MUE = 2.2 MHz and MAX = 7.7 MHz).

**Table 2:** Ground-State Rotational Constants (*A*_0_, *B*_0_, and *C*_0_ in MHz) and ^14^N-Nuclear Quadrupole Coupling Constants (χ in MHz) of the Two Most Stable Phenylalanine Conformers^a^

	IIgg				IIg^–^g			
	^14^N		^15^N		^14^N		^15^N	
param.	exp.^b^	calc.^c^	exp.^b^	calc.^c^	exp.^b^	calc.^c^	exp.^b^	calc.^c^
*A*_0_	1666.0436(14)	1658.3	1646.7381(17)	1639.2	2457.05490(48)	2456.6	2425.69(30)	2425.7
*B*_0_	638.56314(12)	641.3	636.56611(19)	639.4	460.659722(79)	461.1	459.64825(32)	460.2
*C*_0_	568.76843(15)	571.1	569.83617(20)	568.3	424.74604(13)	424.5	423.71892(25)	423.6
χ_*aa*_	–0.283(16)	–0.403			–0.777(22)	–0.505		
χ_*bb*_	1.275(55)	1.479			0.0695(36)	–0.112		
χ_*cc*_	–0.992(39)	–1.076			0.7075(14)	0.617		

a Rotational constants of the ^15^N isotopomers are also reported.

b From ref (23).

c rDSD-LRA equilibrium geometries, rDSD properties and B3 vibrational corrections.

Despite the accessible relative energies of conformers of types I and I′′ (especially Igg), none of them has been detected in the MW study of ref (23), contrary to the conclusions issued from electronic and IR spectra.^10,19^ As a matter of fact, the laser energy needed to obtain MW spectra is much higher than that required by other spectroscopic techniques. This leads to an increased photofragmentation of Phe, with this explaining the weakness of the observed spectra.^23^ At the same time, both theory and experiments agree in indicating that conformers of type II have higher ionization energies than their I and I′ counterparts.^20^ Thus, if the non observed conformers are preferentially ionized in the laser ablation process, their populations in the supersonic jet would be much lower than those estimated from a Boltzmann distribution.

**Figure 3:**
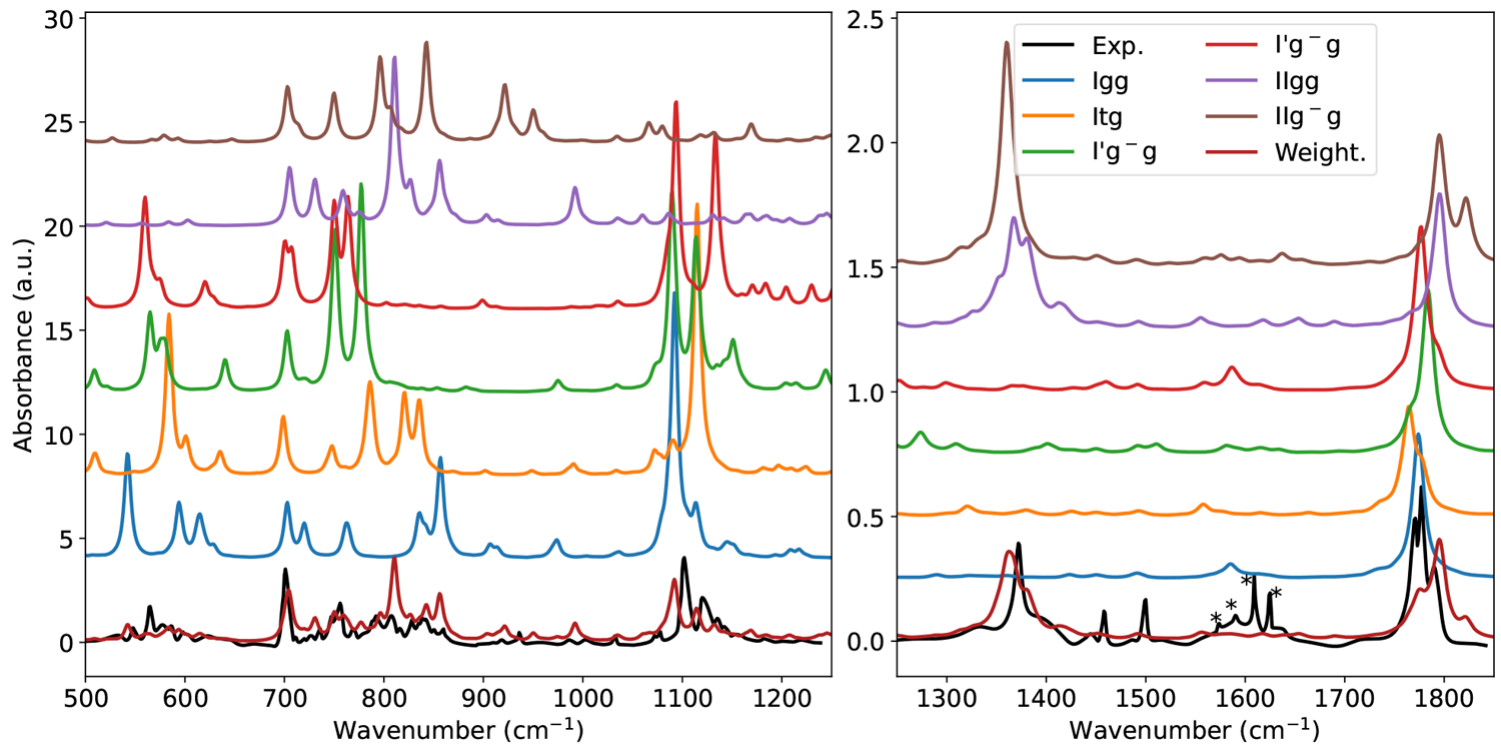
Comparison between the FT-IR spectrum of matrix-isolated phenylalanine (Exp) and the computed anharmonic spectra for the six most stable conformers and their Boltzmann average (Weight.) in the 500–1250 cm^–1^ and 1250–1800 cm^–1^ regions. See main text for details.

Based on these premises, the IR spectra in inert matrices should show the signatures of conformers of types I, I′, II, and, possibly, III. However, in the most relevant spectral regions (mainly OH, C=O and C–O stretching together with COH bending regions) conformers of type III are predicted to absorb at frequencies very close to those of the corresponding type II structures. Therefore, they cannot be characterized by this technique too. Computed and experimental spectra in the mid-IR region are compared in Figure 3. The general agreement between theory and experiment confirms that different conformers are present in the experimental sample and that their relative populations are correctly predicted by our computations. The most distinctive features of the spectra concern the regions of C–O (1060–1160 cm^–1^) and C=O (1750–1800 cm^–1^) stretchings. The presence of multiple bands in the former region proves that several conformers of type I and I′ are present in the matrix. In the latter region, the bands originating from conformers of type I and I′ fall at lower wave numbers than those of type II conformers due to the involvement of the O=C group in a hydrogen bond with the amine hydrogens. However, this kind of hydrogen bond is not very strong, so that the wavenumber shift is quite limited.

The NH stretching vibrations are observed in the experimental spectrum as two broad bands of quite low intensity, centered at about 3340 and 3410 cm^–1^, and the computed values are in good agreement with this finding for all low-lying conformers (see Table 3). In the case of I and I′ conformers these vibrations correspond to symmetric and antisymmetric combinations of the amine NH stretching modes. However, for conformers of type II, the band above 3400 cm^–1^ is due to the antisymmetric NH_2_ stretching, but the band at about 3330 cm^–1^ can be assigned to the out-of-phase combination of NH and OH stretchings. The broad experimental band centered at 3178 cm^–1^ is assigned to the in phase combination of NH and OH stretchings of conformers of type II, which is computed slightly above 3200 cm^–1^. Finally, the OH stretching of conformers of type I and I′ is computed between 3564 and 3579 cm^–1^ and found experimentally between 3557 and 3567 cm^–1^. In more general terms, a remarkable quantitative agreement with experiment is obtained for the positions of all the computed IR bands without employing any adjustable parameter.

**Table 3:** Computed Wavenumbers for the NH and OH Stretching Vibrations of Phenylalanine and Tyrosyne Low-Energy Conformers^a^

phenylalanine				
assign.	Igg	Itg	I′g^–^g	I′*g^–^g
νNH_*sym*_	3325.7	3318.1	3340.8	3364.7
νNH_*asym*_	3403.9	3406.6	3421.7	3444.3
νOH	3572.1	3563.6	3572.6	3579.3
	IIgg	IIg^–^g		
νNH_*sym*_ + νOH in phase	3217.8	3268.8		
νNH_*sym*_ + νOH out of phase	3339.7	3325.9		
νNH_*asym*_	3427.6	3412.0		

tyrosine				
assign.	IICgg	IINgg	IICg^–^g	IINg^–^g
νNH_*sym*_ + νOH in phase	3208.5	3213.6	3243.9	3255.9
νNH_*sym*_ + νOH out-of-phase	3338.6	3337.5	3364.5	3364.5
νNH_*asym*_	3428.1	3426.6	3409.9	3408.9
νOH phenol	3652.3	3661.0	3659.3	3661.1

a See main text for details.

**Figure 4:**
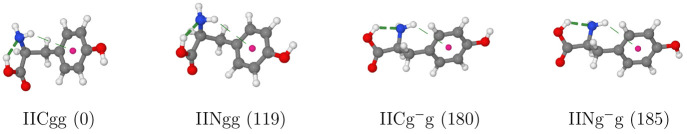
Conformers of tyrosine with the computed relative free energies at room temperature (in cm^–1^) given in parentheses. H-bonds are highlighted by dashed lines.

The six Phe conformers discussed above give rise to 12 conformers of tyrosine (Tyr) due to the already mentioned presence of two nonequivalent orientations of the OH group in the para position of the phenyl ring. All these conformers are true energy minima, but, for the same reasons discussed above for Phe, we can expect that only species of type II are detected in MW experiments (see Figure 4). The structural and energetic characteristics of those four low-energy conformers are collected in Table 4, whereas the different contributions to the final electronic energies are given in Table S4. The situation envisaged by our computations was indeed confirmed by MW experiments, but, unfortunately, for conformers IINg^–^g and IICg^–^g too few lines could be assigned to allow an unequivocal characterization.^24^

**Table 4:** rDSD Relative Electronic Energies (Δ*E*_*rDSD*_), Harmonic Zero Point Energies (ΔZPE_*H*_), Thermal Contributions (ΔTh_*H*_), and Quasi-Harmonic Corrections to Free Energies (*T*Δ*S*_*QH*–*H*_), Together with Differences between junChS-F12 and rDSD Electronic Energies (ΔChS) and B3 Anharmonic Corrections to ZPEs (ΔZPE_*anh*–*H*_) for the Low-Lying Conformers of Tyrosine^a^

label	Δ*E*_*rDSD*_	ΔChS	ΔZPE_*H*_	ΔTh_*H*_	ΔZPE_*anh*–*H*_	*T*Δ*S*_*QH*–*H*_	Δ*G*°^b^	ϕ′	ψ	ω	χ_1_	χ_2_
IICgg^c^	0.0	0.0	0.0	0.0	0.0	0.0	0.0	–26.32	12.33	–2.55	53.08	80.97
IINgg^d^	117.5	3.3	–6.8	–9.5	10.7	4.0	119.2	–26.18	12.34	–2.64	52.72	80.42
IICg^–^g^c^	278.0	11.5	–32.9	–101.9	–10.4	35.6	179.9	33.92	–18.25	4.09	–62.64	104.25
IINg^–^g^d^	275.7	10.8	–37.3	–99.7	–0.9	36.7	185.3	34.06	–18.33	4.08	–63.05	102.84

a Best estimates of relative free energies at room temperature (Δ*G*°) and dihedral angles optimized at the rDSD level (ϕ′, ψ, ω, χ_1_, and χ_2_) are also given. All the energetic quantities are in cm^–1^ and the angles in degrees.

b Sum of columns 2–7.

c χ_3_ = 0°.

d χ_3_ = 180°.

We are thus left with the IINgg and IICgg conformers, whose computed spectroscopic parameters are compared to their experimental counterparts in Table 5 (see also Table S5). For purposes of completeness, the computed spectroscopic parameters of conformers IINg^–^g and IICg^–^g are given in the same tables. The agreement between experimental and computed rotational constants is remarkable, and even more importantly, all the trends and differences between the two conformers are reproduced quantitatively. The percentage MUE for the rotational constants is 0.38% and the percentage MAX 0.56%. These values are comparable to those obtained for the IIgg conformer of Phe and are more than three times smaller than those issued from the MP2 computations reported in ref (24) (percentage MUE = 1.42%, percentage MAX = 1.88%). Larger errors affect the quadrupolar coupling constants, but the agreement between computed and experimental values is largely sufficient to allow an unbiased assignment of different conformers.

**Table 5:** Ground-State Rotational Constants (*A*_0_, *B*_0_, and *C*_0_ in MHz), ^14^N-Nuclear Quadrupole Coupling Constants (χ in MHz), and Electric Dipole Moment Components (μ in debye) of the Two Observed Tyrosine Conformers^a^

parameter	IINgg_exp_^b^	IINgg_calc_^c^	IICgg_exp_^b^	IICgg_calc_^c^	IINg^–^g_calc_^c^	IICg^–^g_calc_^c^
*A*_0_	1529.6791(40)	1522.2	1525.2543(29)	1517.6	2400.9	2404.3
*B*_0_	463.94021(32)	464.5	465.48173(25)	467.7	341.0	340.7
*C*_0_	425.76168(40)	427.1	427.31023(27)	429.4	321.1	321.6
χ_*aa*_	0.709(14)	0.5819	0.740(15)	0.6111	–0.5451	–0.5301
χ_*bb*_	0.236(88)	0.3022	0.247(92)	0.3134	–0.1324	–0.2353
χ_*cc*_	–0.945(88)	–0.8832	–0.988(92)	–0.9245	0.6775	0.7654
μ_*a*_		2.0713		1.4039	4.6739	4.7805
μ_*b*_		5.7026		3.3171	–0.7791	–3.0797
μ_*c*_		0.9601		0.4933	2.1722	1.2795

a The spectroscopic parameters computed for the IINg^–^g and IINg^–^g conformers are also reported.

b From ref (24).

c rDSD-LRA equilibrium geometries, rDSD properties and B3 vibrational corrections.

**Figure 5:**
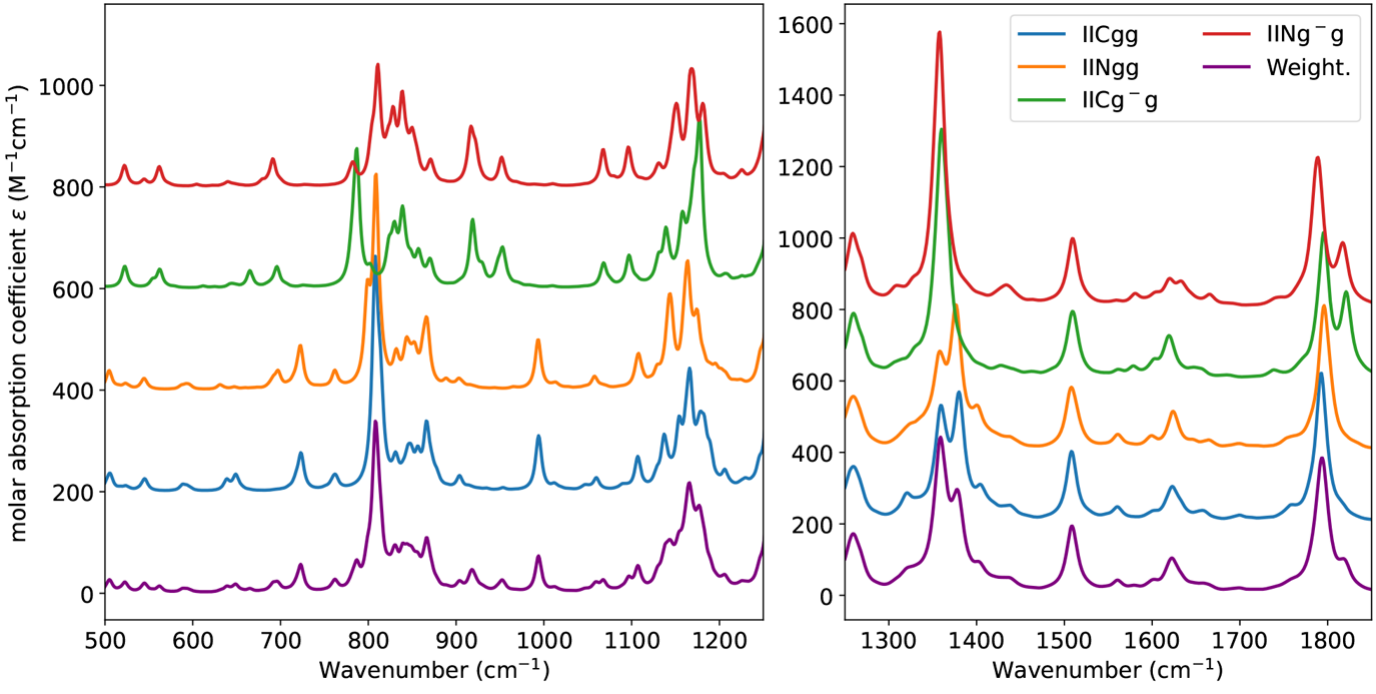
Computed anharmonic spectra for the four conformers and their Boltzmann average (Weight.) in the 500–1250 cm^–1^ and 1250–1800 cm^–1^ regions. See main text for details.

The spectra of tyrosine in the mid-IR region computed taking into account all the type II conformers are shown in Figure 5. Although experimental spectra would probably show some characteristic signatures of conformers of types I and I′ (as discussed above for phenylalanine), in the present context we prefer to avoid any reference to species not well characterized experimentally.

The spectra are very similar to those of the corresponding conformers of phenylalanine except for the presence of a quite intense band at about 1200 cm^–1^ (1197, 1193, 1198, and 1194 cm^–1^ for conformer IICgg, IINgg, IICgg^–^, and IINgg^–^, respectively) assigned to the bending of the phenyl OH moiety. The same trend is observed also in the NH/OH stretching region, where an additional band due to the phenol OH stretching is found at about 3650 cm^–1^ (see Table 3). A more detailed analysis is not pursued because it would be very similar to that given above for phenylalanine.

## Conclusions

In this paper, a general strategy aimed to the unbiased disentanglement of the conformational bath of flexible biomolecule building blocks has been applied to prototypical α-amino acids containing aromatic side chains. Accurate structures and relative energies are obtained by the rDSD-LRA approach and the junChS-F12 composite method, respectively. Next, the spectroscopic parameters of sufficiently populated conformers can be safely computed at the rDSD level. The results obtained for phenylalanine and tyrosine are in full agreement with the available spectroscopic data, and permit their unbiased interpretation in terms of the cooperation or competition between intrabackbone and backbone-(side chain) hydrogen bonds.

Together with the intrinsic interest of the studied molecules, the results of the present investigation show that highly reliable analysis of structural and spectroscopic features is today possible for flexible building blocks of biomolecules in the gas phase.

## Supporting Information Available

Additional computed data for phenylalanine and tyrosine, cartesian coordinates of rDSD optimized geometries for all the low-energy conformers of phenylalanine and tyrosine (PDF)
