# Supplementary material for: Accurate Structures and Spectroscopic Parameters of Phenylalanine and Tyrosine in the Gas Phase: A Joint Venture of DFT and Composite Wave-Function Methods
Source: J Phys Chem A. 2023 Apr 13;127(16):3648–57. doi: 10.1021/acs.jpca.3c01174 (PMC10150396; doi:10.1021/acs.jpca.3c01174)
Supplement: Supplementary file 1 — jp3c01174_si_001.pdf [file jp3c01174_si_001.pdf]

Supporting Information: Accurate Structures and Spectroscopic Parameters of Phenylalanine and Tyrosine in the Gas-Phase: a Joint Venture of DFT and Composite Wave-Function Methods

Vincenzo Barone*,† and Marco Fusè‡

†*Scuola Normale Superiore di Pisa, piazza dei Cavalieri 7, 56126 Pisa, Italy*

‡*DMMT-sede Europa, Università di Brescia, viale Europa 11, 25121 Brescia, Italy*

E-mail: vincenzo.barone@sns.it

Table S1: Relative energy for the low energy conformers of phenylalanine (in cm^{-1}; 1 kJ/mol = 83.59 cm^{-1}) computed at the corresponding rDSD optimized geometries. All the computational levels include the F12 ansatz. CBS and CV contributions are evaluated the the MP2F12 level.

Label	CC/TZ	MP2/TZ	MP2/QZ	CBS	CBS+CV
Igg	311.35	379.63	374.06	301.72	312.75
Itg	631.98	747.24	739.71	618.96	633.44
I'g^-g	653.38	790.50	781.73	638.19	649.41
I'g^-g	720.88	869.01	860.29	705.79	714.44
IIgg	0.00	0.00	0.00	0.00	0.00
IIg^-g	185.37	234.57	232.88	182.44	184.34

Table S2: Equilibrium rotational constants for low-energy phenylalanine conformers obtained at the rDSD level (rDSD) and also including linear regression corrections (rDSD-LRA) together with vibrational corrections at the B3 level of theory. All the values are in MHz.

	rDSD			rDSD-LRA			B3DZ		
	A_e	B_e	C_e	A_e	B_e	C_e	$\Delta_{vib}A$	$\Delta_{vib}B$	$\Delta_{vib}C$
Igg	1605.23	668.38	583.34	1612.96	671.40	585.95	10.1160	4.9120	4.6550
Itg	1734.49	622.25	559.41	1742.85	625.08	561.93	5.8620	4.4480	2.9960
I'g^-g	2430.55	464.43	424.74	2442.53	466.49	426.66	19.5690	3.3850	2.7080
I'g^-g	2477.78	460.99	422.15	2489.95	463.04	424.06	28.078	3.243	2.922
IIgg	1664.80	641.69	571.14	1672.83	644.58	573.69	14.5240	3.3160	2.6140
IIg^-g	2463.95	463.01	426.00	2476.02	465.08	427.93	19.4310	3.9680	3.4330

Table S3: ^{14}N nuclear quadrupole coupling constants (χ, in MHz) and dipole moment components (μ, in debye) of low-energy conformers of phenylalanine from rDSD computations.

	χ_{aa}	χ_{bb}	χ_{cc}	μ_a	μ_b	μ_c
Igg	1.5345	-2.4484	0.9139	-0.27	-0.79	-0.90
Itg	2.7114	-4.4535	1.7420	0.42	0.39	1.29
I'g$^-$g	0.5862	2.7579	-3.3442	1.56	1.24	-1.01
I'g$^-$g	1.7240	1.6166	-3.3406	-0.88	1.57	-0.69
IIgg	-0.4033	1.4790	-1.0757	2.13	4.39	0.31
IIg$^-$g	-0.5051	-0.1115	0.6165	4.59	-2.00	1.90

Table S4: Relative energy for the low energy conformers of tyrosine (in cm^{-1}; 1 kJ/mol = 83.59 cm^{-1}) computed at the corresponding rDSD optimized geometries. All the computational levels include the F12 ansatz. CBS and CV contributions are evaluated the the MP2F12 level.

Label	CC/TZ	MP2/TZ	MP2/QZ	CBS	CBS+CV
IICgg	0.00	0.00	0.00	0.00	0.00
IINgg	121.02	124.58	123.74	119.56	120.76
IICg$^-$g	291.30	331.02	328.16	286.35	289.45
IINg$^-$g	287.96	325.27	322.79	283.68	286.56

Table S5: Equilibrium rotational constants for low-energy tyrosine conformers obtained at the rDSD level (rDSD) and also including linear regression corrections (rDSD-LRA) together with vibrational corrections at the B3 level of theory. All the values are in MHz.

	rDSD			rDSD-LRA			B3DZ		
	A_e	B_e	C_e	A_e	B_e	C_e	$\Delta_{vib}A$	$\Delta_{vib}B$	$\Delta_{vib}C$
IINgg	1529.03	466.15	427.72	1535.67	468.02	429.41	13.62	3.50	2.31
IICgg	1524.69	467.69	429.28	1531.35	469.57	430.98	13.74	1.86	1.55
IINg$^-$g	2410.88	342.30	322.22	2421.50	343.66	323.51	20.56	2.63	2.37
IICg$^-$g	2412.96	341.98	322.64	2423.57	343.35	323.94	19.26	2.68	2.36

rDSD Geometries

Phenylalanine – Igg

```
23
Enegy:   -554.00298806
C             2.213600      -1.477023       0.037105
C             1.021587      -1.086910      -0.568431
C             0.700806       0.266747      -0.712758
C             1.607532       1.222695      -0.243615
C             2.800918       0.835842       0.362950
C             3.106232      -0.515907       0.507225
H             2.447135      -2.530462       0.138140
H             0.334963      -1.839453      -0.941534
C            -0.610035       0.685223      -1.329100
H             1.374034       2.275617      -0.358359
H             3.493139       1.590003       0.718822
H             4.034757      -0.817941       0.976714
C            -1.738809       0.834047      -0.286976
N            -1.417699       1.878191       0.667419
C            -2.047316      -0.507903       0.369112
O            -1.959973      -0.751330       1.547347
O            -2.463305      -1.426744      -0.537980
H            -0.501584       1.656161      -1.815885
H            -2.176060       1.978709       1.333169
H            -0.610291       1.593937       1.214061
H            -2.651617      -2.235170      -0.038641
H            -0.920546      -0.041446      -2.081683
H            -2.644227       1.124285      -0.829604
```

Phenylalanine – Itg

```
23
Enegy:   -554.00171436
C             2.752502      -0.863405       0.776126
C             1.479601      -1.321934       0.448267
C             0.689189      -0.637682      -0.479856
C             1.202516       0.517133      -1.075543
C             2.476469       0.979875      -0.750147
C             3.254504       0.291387       0.177574
H             3.353906      -1.408064       1.494595
H             1.095511      -2.224262       0.913562
C            -0.706262      -1.112733      -0.791272
H             0.598573       1.056853      -1.797353
H             2.861458       1.875222      -1.224269
H             4.246131       0.648123       0.429234
C            -1.740178      -0.651722       0.249579
N            -3.056239      -1.152585      -0.130879
C            -1.697754       0.868748       0.332314
O            -2.092643       1.607018      -0.540823
O            -1.167775       1.315152       1.489253
```

H	-1.022691	-0.735821	-1.768231
H	-3.737950	-0.942759	0.590210
H	-3.364642	-0.671801	-0.970813
H	-1.146296	2.282657	1.429848
H	-0.750997	-2.203195	-0.824401
H	-1.476090	-1.059418	1.226785

Phenylalanine – I'g^{-}g

23
Enegy: -554.00142491

C	3.089416	-1.056336	-0.249210
C	1.741556	-1.038328	-0.607846
C	0.981413	0.126318	-0.476439
C	1.601380	1.277106	0.022549
C	2.945920	1.264070	0.381204
C	3.694525	0.094744	0.247643
H	3.664591	-1.967956	-0.361102
H	1.276763	-1.935558	-1.002965
C	-0.485413	0.137799	-0.811512
H	1.025549	2.191456	0.125281
H	3.411284	2.165795	0.761632
H	4.741693	0.083945	0.525011
C	-1.356827	0.009587	0.468146
N	-1.157387	-1.202428	1.241644
C	-2.803947	0.065768	0.031775
O	-3.494103	-0.896762	-0.202847
O	-3.231850	1.337023	-0.135029
H	-0.746541	1.068427	-1.323794
H	-1.585771	-1.987450	0.762244
H	-0.167244	-1.393269	1.345257
H	-4.139377	1.279294	-0.469336
H	-0.728496	-0.694519	-1.478617
H	-1.156870	0.877280	1.101608

Phenylalanine – I'*g^{-}g

23
Enegy: -554.00096146

C	-3.064829	-1.019716	0.427430
C	-1.724708	-0.893298	0.790026
C	-0.994962	0.245568	0.439200
C	-1.637349	1.256519	-0.283005
C	-2.975669	1.133814	-0.648103
C	-3.693911	-0.007223	-0.293711
H	-3.617239	-1.908277	0.710165
H	-1.235450	-1.684690	1.346505
C	0.466262	0.370831	0.786212
H	-1.085387	2.151085	-0.554486
H	-3.458959	1.929591	-1.202891

H	-4.736213	-0.103545	-0.573361
C	1.350532	-0.103600	-0.381191
N	1.106252	-1.508604	-0.663575
C	2.827213	0.079077	-0.090011
O	3.686833	-0.739370	-0.310399
O	3.099415	1.306362	0.409155
H	0.707603	1.409373	1.017713
H	0.235880	-1.621778	-1.167820
H	1.861064	-1.891070	-1.220788
H	4.060766	1.344950	0.520397
H	0.700554	-0.240747	1.660812
H	1.150348	0.560628	-1.238348

Phenylalanine – IIgg

23
Enegy: -554.00443396

C	2.135046	-1.558849	0.083211
C	0.967648	-1.089922	-0.513491
C	0.760298	0.284004	-0.687753
C	1.752673	1.170333	-0.256724
C	2.922603	0.702553	0.341162
C	3.114621	-0.665366	0.515161
H	2.281277	-2.625259	0.208246
H	0.210781	-1.789253	-0.849062
C	-0.523565	0.804421	-1.283414
H	1.615734	2.236980	-0.406348
H	3.682340	1.404951	0.663573
H	4.022693	-1.033685	0.977189
C	-1.662443	0.875746	-0.249010
N	-1.345678	1.605057	0.983531
C	-2.171674	-0.533419	0.103147
O	-2.331615	-1.398405	-0.722723
O	-2.461281	-0.701906	1.396911
H	-0.361646	1.808315	-1.684824
H	-0.358574	1.513515	1.205377
H	-1.549377	2.591712	0.893254
H	-2.222569	0.152785	1.817324
H	-0.855877	0.162725	-2.101001
H	-2.520507	1.367700	-0.717445

Phenylalanine – IIg^{-}g

23
Enegy: -554.00362797

C	3.064521	-1.079678	-0.318772
C	1.718287	-1.011290	-0.679520
C	0.980330	0.156597	-0.472471
C	1.620206	1.261109	0.100939
C	2.962661	1.197619	0.460687

C	3.688993	0.024298	0.254022
H	3.622805	-1.992423	-0.490486
H	1.239242	-1.870333	-1.138647
C	-0.485311	0.219859	-0.815178
H	1.062481	2.178857	0.257962
H	3.445054	2.064035	0.897646
H	4.734817	-0.024462	0.532199
C	-1.372337	0.126751	0.439587
N	-1.186659	-1.168826	1.099431
C	-2.828457	0.278605	-0.010253
O	-3.307419	1.338920	-0.323827
O	-3.507113	-0.876614	-0.071310
H	-0.725137	1.158339	-1.318777
H	-1.407918	-1.107337	2.086853
H	-0.218967	-1.465639	1.031631
H	-2.882979	-1.553914	0.264354
H	-0.747919	-0.598622	-1.492200
H	-1.149064	0.979353	1.087799

Tyrosine – IICgg

24
Enegy: -629.14888686

C	2.130979	-1.572394	0.079087
C	0.967610	-1.093153	-0.515287
C	0.758711	0.279514	-0.686943
C	1.759388	1.154203	-0.249624
C	2.927626	0.688986	0.346996
C	3.111690	-0.681348	0.515022
H	2.276047	-2.641361	0.200691
H	0.212999	-1.794920	-0.850777
C	-0.522863	0.805872	-1.281638
H	1.632437	2.222985	-0.392496
H	3.701047	1.371096	0.677457
C	-1.664087	0.876779	-0.250260
N	-1.348861	1.608759	0.981306
C	-2.166280	-0.533551	0.105957
O	-2.320326	-1.403144	-0.716524
O	-2.454737	-0.699877	1.400384
H	-0.357560	1.811112	-1.678702
H	-0.361160	1.518032	1.201403
H	-1.553240	2.595095	0.888770
H	-2.220314	0.157855	1.817526
H	-0.855678	0.170651	-2.104157
H	-2.524060	1.365035	-0.719305
O	4.273124	-1.094139	1.105155
H	4.273806	-2.055334	1.158030

Tyrosine – IINgg

```
24
Enegy:   -629.14835160
C              2.142857      -1.552265       0.096916
C              0.978741      -1.079826      -0.494502
C              0.763266       0.293272      -0.680387
C              1.760028       1.174574      -0.257578
C              2.934547       0.714122       0.337988
C              3.123953      -0.653597       0.517759
H              2.307872      -2.613490       0.235464
H              0.223960      -1.785727      -0.821053
C             -0.521261       0.808547      -1.278438
H              1.630861       2.241815      -0.409088
H              3.699438       1.417433       0.652168
C             -1.666305       0.873556      -0.250850
N             -1.357034       1.604009       0.983142
C             -2.168212      -0.538316       0.099653
O             -2.318487      -1.405019      -0.726331
O             -2.462423      -0.708515       1.392310
H             -0.362358       1.814147      -1.677293
H             -0.369405       1.516085       1.204703
H             -1.564584       2.589813       0.891914
H             -2.230023       0.147439       1.813586
H             -0.847067       0.169068      -2.100535
H             -2.525476       1.360961      -0.722213
O              4.249358      -1.175096       1.091441
H              4.845043      -0.456558       1.326709
```

Tyrosine – IICg^{-}g

```
24
Enegy:   -629.14762038
C              3.069897      -1.092742      -0.334477
C              1.722482      -1.015079      -0.689535
C              0.981115       0.145994      -0.471905
C              1.630996       1.240294       0.113427
C              2.970349       1.179892       0.471617
C              3.693800       0.006463       0.248782
H              3.630022      -2.004238      -0.517866
H              1.249121      -1.873547      -1.155264
C             -0.483974       0.216087      -0.813877
H              1.079945       2.159634       0.283872
H              3.471909       2.030035       0.917002
C             -1.375512       0.134244       0.438900
N             -1.187918      -1.154643       1.111047
C             -2.830111       0.277934      -0.016700
O             -3.312574       1.334075      -0.339003
O             -3.504042      -0.880692      -0.073898
H             -0.720271       1.151556      -1.325006
H             -1.414011      -1.085478       2.096889
H             -0.217459      -1.444277       1.050191
H             -2.878947      -1.553007       0.269697
H             -0.750000      -0.604950      -1.486702
```

H	-1.156054	0.993434	1.079739
O	5.008530	0.001959	0.619728
H	5.395277	-0.852225	0.401880

Tyrosine – IINg⁻g

24

Enegy: -629.14763052

C	3.071213	-1.073485	-0.308231
C	1.727543	-0.999796	-0.665007
C	0.980965	0.165193	-0.466123
C	1.625813	1.267631	0.101312
C	2.968102	1.211165	0.461853
C	3.693139	0.035812	0.259129
H	3.646576	-1.976526	-0.469505
H	1.254719	-1.864671	-1.119567
C	-0.484291	0.225414	-0.809472
H	1.074806	2.189400	0.257910
H	3.453001	2.080714	0.894524
C	-1.376679	0.126359	0.441480
N	-1.186736	-1.169539	1.099010
C	-2.831430	0.271932	-0.013123
O	-3.316351	1.331063	-0.321996
O	-3.502420	-0.887344	-0.085394
H	-0.727786	1.162772	-1.313573
H	-1.415748	-1.112991	2.084970
H	-0.215726	-1.457013	1.037391
H	-2.875925	-1.562635	0.249775
H	-0.742754	-0.592570	-1.488904
H	-1.159751	0.978981	1.092014
O	5.012938	-0.082231	0.591639
H	5.321723	0.751855	0.959796
